# Perinatal outcomes of twin pregnancies complicated with preeclampsia at a tertiary hospital in Ethiopia: A case‐control study

**DOI:** 10.1002/puh2.37

**Published:** 2022-11-14

**Authors:** Abraham Fessehaye Sium, Don Eliseo III Lucero‐Prisno, Wondimu Gudu

**Affiliations:** ^1^ Department of Obstetrics and Gynecology St. Paul's Hospital Millennium Medical College (SPHMMC) Addis Ababa Ethiopia; ^2^ Faculty of Management and Development Studies University of the Philippines (Open University) Los Baños Laguna Philippines

**Keywords:** hypertension in twin pregnancy, preeclampsia, prematurity, twin delivery, twin gestation

## Abstract

**Background:**

Preeclampsia accounts for 10–15% maternal deaths globally, corresponding to 50,000 annual maternal deaths. Twin pregnancy is a known risk factor for preeclampsia; however, there is inadequate data on the clinical characteristics and perinatal outcomes of twin pregnancies complicated with preeclampsia. This paper studied the perinatal outcomes of twin pregnancies complicated with preeclampsia at a tertiary hospital in Ethiopia.

**Methods:**

A case‐control study was conducted at St. Paul's Hospital Millennium Medical College (Addis Ababa, Ethiopia) from September 1, 2016 till August 31, 2018. A total of 173 twin deliveries (63 preeclampsia cases and 110 normotensive controls) were included in the study and the primary outcome was the frequency of preterm delivery. Data were analyzed using SPSS version 23 and statistical test of association was done using chi‐square test for categorical data. Variables with *p* value of <0.2 on bivariate analysis were entered into multivariable logistic regression analysis. *p* value <0.05 were considered significant.

**Results:**

The frequency of preterm birth was 61.9% in the preeclampsia group and 33.6% in the normotensive group, *p* < 0.001. Preeclampsia group were 2.58 times more likely to have preterm delivery compared to matched normotensive controls [adjusted OR = 2.58, 95% CI (1.24 – 5.35), *p* = 0.01]. There was no difference in the rate of adverse neonatal outcome (respiratory distress syndrome, early neonatal death, and Low Apgar score) between the groups.

**Conclusions:**

In this study, twin pregnancies complicated with preeclampsia were found to have an increased rate of preterm birth compared to matched controls without hypertension.

## INTRODUCTION

Hypertension in pregnancy complicates 10% of pregnancies [1, 2]. Preeclampsia/Eclampsia is one of the leading causes of maternal morbidity and mortality globally [3]. It remains one of the most common causes of perinatal mortality and morbidity in developing countries, mainly due to high rate of preterm birth associated with it [4], contributing to 500,000 perinatal deaths annually [5].

Preeclampsia (PE) occurs in 6–31% of twin pregnancies [6]. Twin pregnancy is a known risk factor for preeclampsia. However, the reason why it is seen more in twin pregnancies is still yet not known [3, 7]. A 2014 review on the risk factors for preeclampsia in twin pregnancy reported that chorionicity is not associated with the occurrence of preeclampsia [8]. Twin pregnancies also have increased rates of post‐partum hemorrhage and preterm birth [9]. A study among 997 twin gestations showed that preeclampsia in twin pregnancy was associated with increased rate of cesarean delivery, low Apgar score, and lower mean gestational age at delivery [10]. Another study done on the perinatal outcome of twin gestations with preeclampsia concluded that twin pregnancies with PE were associated with worse perinatal outcomes and increased frequency of preterm delivery [11].

Although the incidence of preeclampsia in twin pregnancy has been well studied, there is inadequate data regarding it's perinatal outcomes. This study aimed to determine perinatal outcomes of twin pregnancies complicated with preeclampsia at tertiary hospital in Ethiopia.

## METHODS

### Study design

This is a case‐control study conducted at Saint Paul's Hospital Millennium Medical College (SPHMMC), in Addis Ababa, Ethiopia, from 2016 to 2018. SPHMMC is tertiary teaching hospital and a leading medical college in Ethiopia with various specialty and sub‐specialty level care and training programs. An average of 11,500 deliveries are attended to per annum at the hospital. It's maternal‐fetal medicine sub‐specialty unit provides an inclusive prenatal and maternity care to women with high‐risk pregnancies, such as twin pregnancy complicated with preeclampsia.

### Study population

A total of 423 twin pregnancy records were retrieved from the Hospital Management Information system between September 1, 2016 and August 31, 2018. Out of which, 173 twin pregnancies (63 preeclampsia cases and 110 normotensive cases‐controls) were included in the final analysis based on the inclusion and exclusion criteria.

Delivery registry was used to identify twin pregnancies complicated with preeclampsia and twin without any form of hypertension (normotensive) who had their deliveries attended at SPHMMC. The inclusion criteria were: twin pregnancy with confirmed chorionicity; gestational age (GA) >28 weeks of gestation; alive co‐twin with no congenital anomaly; and antenatal care follow‐up at SPHMMC or its catchment health centers.

The exclusion criteria were: gestational hypertension, chronic hypertension, chronic hypertension with superimposed preeclampsia, eclampsia, unknown date (unreliable date from last normal menstrual period and no early ultrasound), concurrent medical or obstetric illness, and higher order pregnancies.

Controls were matched cases with no hypertension. Normotension was defined as twin pregnancies with no hypertensive disorders of pregnancy and blood pressure in the range of <130/90 mmHg and ≥90/60 mmHg  measured in the right arm. Gestational age was determined from early ultrasound and from reliable last normal menstrual period date in those cases that lacked early ultrasound.

### Data collection and variables

Data were collected by reviewing maternal charts and neonatal intensive care unit (NICU) registry. Sociodemographic data, antenatal care and obstetric care characteristics, and perinatal outcomes were extracted using a structured questionnaire prepared in English language. Data were collected by second year obstetrics and gynecology residents. Completeness and consistency of the data was checked by the principal investigator. The primary outcome of the study was rate of preterm delivery (delivery at gestational age of less than 37 weeks). Secondary outcomes were the rate of intrauterine growth restriction, low Apgar score (1 and 5 min Apgar score of <7), early neonatal death, respiratory distress syndrome, low birth weight, and very low birth weight.

### Data analysis

Data were entered into Epi Info 7, and exported to SPSS version 23 for analysis. Statistical test of association was done using chi‐square for categorical data; strength of association is reported using odds ratio and 95% confidence interval. Those variables with *p* < 0.2 on bivariate analyses were tested using multivariable logistic regression analysis *p* < 0.05 were considered significant.

### Ethics considerations

Ethical clearance was obtained from the Institutional Review Board, SPHMMC. Because it is a retrospective study, informed consent from study participants was not required for this study, hence it was not obtained.

## RESULTS

The frequency of preterm birth in the PE group was statistically different from that observed in the normotensive group (61.9 vs. 33.6%, *p* < 0.001) (Table 1) but the was no difference in rate of early preterm delivery (delivery at <34 weeks) between the groups (12.7 vs. 9.1%, *p* = 0.568).

**TABLE 1:** Baseline characteristics of women with twin pregnancies who delivered a tertiary hospital in Ethiopia, 2016–2018

	Twin pregnancy with normotensive (*n* = 110)		Twin pregnancy with preeclampsia (*n* = 63)		
Parameters	*n*	(%) or (SD)	*n*	(%) or (SD)	*p* value
Maternal age (years), mean ± SD	26.9	(±4.2)	28.27	(±4.6)	0.049
Estimated fetal weight (in grams), mean ± SD	2282	(±434.5)	2120.35	(±436.3)	0.003
Mode of delivery					0.072
Spontaneous vaginal delivery	41	(37.6)	13	(21.0)	
Cesarean section	65	(59.6)	47	(75.8)	
Instrumental delivery	3	(2.8)	2	(3.2)	
Gravidity					
Primigravida	41	(38.0)	18	(28.6)	0.245
Multigravida	67	(62.0)	45	(71.4)	
ANC Status					
Booked at SPHMMC	72	(65.5)	41	(65.08)	0.960
Booked at Catchment HC	38	34.6	22	(34.9)	
Chorionicity					
Monochorionicity	99	(90.0)	58	(92.1)	0.788
Dichorionicity	11	(10.0)	5	(5.9)	
Discordance of EFW≥20%	12	(13.3)	6	(11.3)	0.726
IUGR (EFW less than 5^th^ percentile)	7	(6.5)	7	(10.8)	0.748
Gestational age at delivery					
Preterm delivery (<37 wks)	37	(33.6)	39	(61.9)	<0.001
Early preterm delivery (<34 weeks)	10	(9.1)	8	(12.7)	0.568
Term delivery (≥37 weeks)	73	(66.4)	24	(38.1)	0.001

Abbreviations: ANC, antenatal care; EFW, estimated fetal weight; SD, standard deviation; SPHMMC, St. Paul's Hospital Millennium Medical College.

Among the important findings of this study was the lack of uniformity in the gestational age calculation methods (Table 2). More number of cases in the normotensive group had both early ultrasound findings and reliable last normal menstrual dates available to calculate the gestational age.

**TABLE 2:** Distribution of potential confounders in twin pregnancies complicated with preeclampsia and twin with normotensive pregnancy cases in Ethiopia, 2016–2018

Variables	Normotensive twin pregnancy (*n* = 110)	Twin pregnancy with preeclampsia (*n* = 63)	*p* value
GA calculation			
Both reliable LNMP and early US	17 (15.6)	3 (4.8)	0.012
Early US	61 (56.0)	29 (46.8)	
Only reliable LNMP	31 (28.4)	30 (48.4)	
CS done in labor, *n* (%)	41 (62.1)	11 (22.9)	<0.001
CS or instrumental delivery for fetal distress	4 (6.4)	0 (0.0)	0.128
Dexamethasone treatment, *n* %	1(0.1)	9 (14.3)	0.001

Abbreviations: CS, cesarean section; GA, gestational age; LNMP, last normal menstrual period; US, ultrasound.

Multivariable logistic regression data analysis (Table 3) shows that the adjusted Odds Ratio (adjusted OR) for preterm birth was significantly increased [adjusted OR = 2.58, 95% CI (1.24–5.35), *p* = 0.01]. There were five early neonatal deaths in the PE group while there was only one such early neonatal death in the normotensive group, which was not statistically significant [adjusted OR = 2.98, 95% CI (0.16–55.59), *p* = 0.465]. Similarly, there was no significant difference in occurrence of respiratory distress syndrome between the groups [adjusted OR = 1.47, 95% CI (0.45–4.70), *p*‐value 0.519], nor intra‐uterine growth restriction [adjusted OR = 1.85, 95% CI (0.38–9.01), *p* = 0.44], nor low Apgar score [adjusted OR = 0.96, 95% CI (0.33–2.76), *p* value = 0.933] nor in the rate of low birth weight and very low birth weight babies [adjusted OR = 0.99 (0.43–2.30) *p* = 0.989, and adjusted OR = 0.33 (0.06–1.79), *p* = 0.201, respectively].

**TABLE 3:** Selected perinatal outcomes in twin pregnancy with preeclampsia compared with normotensive group in Ethiopia, 2016–2018

	Normotensive group		Preeclampsia group				
Perinatal outcome	*n*	(%)	*n*	%	OR (95% CI)	Adjusted OR (95% CI)	*p*‐value
Preterm delivery	37	(33.6)	39	(61.9)	3.21 (1.68–6.11)	2.58 (1.24–5.35)	0.011
RDS, *n* (%)	19	(16.5)	22	(30.1)	2.05 (0.85–4.98)	1.47 (0.45–4.70)	0.519
IUGR, *n* (%)	7	(6.5)	7	(10.8)	1.44 (0.42–4.92)	1.85 (0.38–9.01)	0.444
Low ApgarScore, *n* (%)	16	(14.4)	13	(20.0)	1.31 (0.54–3.14)	0.96 (0.33–2.76)	0.933
LBW, *n* (%)	123	(80.4)	78	(85.7)	1.44 (0.69–3.03)	0.99 (0.43–2.30)	0.989
VLBW, *n* (%)	14	(12.2)	7	(11.1)	1.40 (0.50–3.97)	0.33 (0.06–1.79)	0.201
END, *n* (%)	1	(0.9)	5	(7.8)	7.25 (0.79–66.41)	2.98 (0.16–55.59)	0.465

Abbreviations: END, early neonatal death; IUGR, intrauterine growth restriction; LBW, low birth weight; RDS, respiratory distress syndrome; VLVW, very low birth weight.

## DISCUSSION

In this study, twin pregnancies complicated with preeclampsia had an increased rate of preterm birth, compared to those without hypertension. However there was no difference in the rate of early preterm birth (birth at less than 34 weeks) between the groups. Similarly, there was no difference in the rate of adverse neonatal outcome (respiratory distress syndrome, early neonatal death, and Low Apgar score) between the groups.

Multiple studies show that twin gestations complicated by preeclampsia have a higher rate of preterm delivery when compared to singletons. However, it is not known whether this is the result of increased incidence of preterm labor in twins or as a result of the difference in the clinical behavior of preeclampsia in these two different obstetric populations [11, 12, 13]. There are too few studies that report on the course of preeclampsia in twin pregnancies in terms of preterm birth. The higher frequency of preterm birth observed in the preeclampsia group in our study remained unchanged after controlling known contributors to preterm delivery. This finding is consistent with a report of a recent study conducted in China, in which perinatal outcomes of 143 preeclamptic women were compared with 367 normotensive women with twin pregnancies. The study found that twin pregnancies complicated with preeclampsia were 2.75 more likely to have preterm birth compared to controls [14]. Likewise, a cohort study from Israel which compared perinatal outcomes of 19 cases of severe preeclampsia to 44 matched controls reported that preeclampsia in twin gestation was associated with increased rate of preterm birth. The mean gestational age at delivery was 34.8 ± 3.5 weeks in the severe preeclampsia cases compared to 36.2 ± 2.8 weeks in the controls [15].

The finding of high preterm birth rate among women with twin pregnancy complicated with preeclampsia in our study implies institution of pre‐delivery obstetric interventions aiming at alleviating complications of preterm birth that should synchronize with readiness for the management of preterm neonates. However, this warrants further exploration as important limitations overshadow these interpretations. The main limitations of this study are retrospective data collection, small sample size allocation, the finding of no difference in the rate of preterm delivery at gestational age less than 34 weeks, and non‐uniformity of gestational age calculation methods. The lack of random sampling technique is the other limitation. We recommend a further analytic study, preferably prospective comparative study, with appropriate sample size allocation, ideal and uniform gestational age calculation method, correct sub‐group analysis such as spontaneous versus indicated preterm delivery and analysis of adverse maternal outcomes.

## CONCLUSION

In this study, twin pregnancies complicated with preeclampsia were found to be associated with high preterm birth rate. Although this may imply readiness for pre‐delivery and post‐delivery interventions to alleviate complications of preterm delivery in such pregnancies, it warrants further exploration and we recommend further analytic study.

## AUTHOR CONTRIBUTIONS

Wondimu Gudu and Abraham Fessehaye Sium developed the concept and design of study and subsequent data collection and analysis. Abraham Fessehaye Sium, Wondimu Gudu, and Don Eliseo III Lucero‐Prisno drafted the manuscript and edited the final manuscript. All authors critically revised the article for intellectual content and gave final approval.

## CONFLICTS OF INTEREST

The authors declare that they have no financial or non‐financial competing interests. Abraham Fessehaye Sium is an Editorial Board member of Public Health Challenges and co‐author of this article. Don Eliseo III Lucero‐Prisno is the Editor‐in‐Chief of Public Health Challenges and co‐author of this article. They were excluded from editorial decision‐making related to the acceptance of this article for publication in the journal.

## FUNDING INFORMATION

No funding was received for this study.

## ETHICS APPROVAL

Ethical clearance & permission letter was obtained from the Institutional Review Board (IRB) of St. Paul's hospital millennium Medical College (SPHMMC). A written informed consent was not required for this study; therefore, not obtained.

## Data Availability

The datasets generated and analyzed in this study are available from the corresponding author on reasonable request.
